# Effect of straw and inhibitors on the fate of nitrogen applied to paddy soil

**DOI:** 10.1038/s41598-020-78648-w

**Published:** 2020-12-09

**Authors:** Chunxiao Yu, Xueshi Xie, Hengzhe Yang, Lijie Yang, Wentao Li, Kaikuo Wu, Weiming Zhang, Chen Feng, Dongpo Li, Zhijie Wu, Lili Zhang

**Affiliations:** 1 Institute of Applied Ecology, Chinese Academy of Sciences, Shenyang, 110016 People’s Republic of China; 2 University of Chinese Academy of Sciences, Beijing, 100049 People’s Republic of China; 3 Stanley Agriculture Group Co., Ltd, Linshu, 276700 People’s Republic of China; 4 Shenyang Research Institute of Chemical Industry, Shenyang, 110021 People’s Republic of China; 5 Biochar Engineering and Technology Research Center of Liaoning Province, Shenyang Agriculture University, Shenyang, 110866 People’s Republic of China; 6 Tillage and Cultivation Research Institute, Liaoning Academy of Agricultural Sciences, Shenyang, 110161 People’s Republic of China

**Keywords:** Element cycles, Biogeochemistry

## Abstract

A pot experiment was used to explore the distribution of fertilizer N and agronomic effects in a paddy soil-rice (*Oryza sativa* L.) system. Five treatments were set: without nitrogen, straw and inhibitor (C), urea (U), urea + straw (US), urea + urease + nitrification inhibitor (UI) and urea + urease + nitrification inhibitor + straw (UIS). Soil and urea-derived microbial biomass N increased significantly in US and UIS compared with straw-free treatments at seedling and tillering, indicating that biotic process play an important role in the retention of fertilizer N with straw addition. About 10% urea-N was recovered as fixed ammonium (FA) at seedling stage, subsequently released at tillering and maturation regardless of treatments, which emphasizes the importance of FA in the retention and supply of fertilizer N in paddy soils. Compared with U, rice yield and N uptake in US decreased by 7.8% and 25.2% respectively, while inhibitors (UIS) alleviated the reduction by 16.4% and 31.6%. The current research indicated UIS is recommended as the most appropriate management strategy in paddy soils of Northeast China based on N dynamics. But the economic effect as well as the field-scale validation need to be further evaluated.

## Introduction

Rice (*Oryza sativa* L.) cultivation in China covers about 3.02 × 10^7^ ha, and overall yield reached 2.07 × 10^8^ t in 2018, accounting for 50% of Chinese grain production^1^. As the most important nutrient element, an adequate supply of nitrogen (N) contributes to more than half of the rice yield^2^. Over-application of N, however, results in low use efficiency and high N losses, and causes environmental problems^3^. An input of fertilizer N can be absorbed by rice directly or temporarily immobilized by biotic and abiotic processes and subsequently released to meet crop demand. These processes are influenced by different management practices such as the addition of urease and nitrification inhibitors, and return of organic materials^4,5^.

Improving immobilization of fertilizer N is an effective way to retain N and reduce N losses with the addition of decomposable carbon (C)^6^. Previous studies have indicated that available C inputs temporarily enhance microbial immobilization of N, resulting in an increase in soil microbial nitrogen (MBN)^7^; this process is closely correlated with biotic processes. Ma et al.^7^ indicated that MBN consistently increased with inputs of the nitrification inhibitor DMPP (3,4-dimethylpyrazole phosphate), whereas the urease inhibitor NBPT (N-butyl thio-phosphotriamine) increased MBN in the absence of glucose but decreased it in the presence of glucose. Rice paddy soils show a different response pattern due to flooded conditions: Wang et al.^8^ found that flooded soil has a lower immobilization rate of N into microbial biomass because of the loss of ammonium N from volatilization and/or greater NO_3_^−^ leaching after nitrification, but straw return generally stimulates immobilization^9–11^, and consequently changes the way fertilizer N is conserved and supplied^12–14^. With the addition of urease inhibitor and nitrification inhibitor, the forms of inorganic N are regulated in soil, and under flooded conditions, the persistence of fertilizer NH_4_^+^ influences the retention and distribution pattern of N in soil, especially combined with the application of straw. Currently, however, little information is available regarding the scenarios mentioned above.

Fertilizer NH_4_^+^ can also be rapidly fixed by soil clay minerals, especially 2:1 clay minerals. More than 70% of applied fertilizer N can be retained as fixed NH_4_^+^ in some arable soils^15,16^. Fixed ammonium (FA) is an active abiotic N pool and more than 80% of recently FA can be released in subsequent growing seasons^17^. Increasing fixed NH_4_^+^ is another way to build a pool of N that will later become available to crops, improving fertilizer recovery and minimizing N losses^18^. In flooded paddy soils, how the FA pool responds to the addition of inhibitors and straw remains to be evaluated.

Although the response of crop yield and N uptake to inhibitors is variable, a meta-analysis concluded that the addition of inhibitors caused the yield and N use efficiency to increase by an average of 7.5% and 12.9%, respectively^19^. The greater persistence or continuous supply of NH_4_^+^ with the addition of nitrification and urease inhibitors was reported and responsible for the improved agronomic effect. Other research indicated that declining yield was found in continuously cropped irrigated rice systems, where a significant portion of the rice residue is returned^3^. An inadequate N supply in the late season was suggested to be responsible for the declining yield when rice residue is returned^20^. Eagle (2000) also indicated that the decrease in N uptake does not result from a reduction in the capacity of the root system to acquire N from soil^20^. The strong retention of fertilizer N in different soil N pools with the application of straw is probably responsible for the inadequate supply of N to rice. It is therefore necessary to evaluate the fate of fertilizer N and its retention in different pools, with the concomitant use of inhibitors and straw application, and observe how this retention affects its uptake by rice.

Based on the analysis above, a pot experiment was set up to clarify the impacts of inhibitor and straw additions on the dynamics of fertilizer N in a paddy soil-rice system by using ^15^N to trace the fate of applied N. We hypothesized that: (1) Straw decreases N uptake and crop yield, stimulates microbial growth and increases the ability of organic nitrogen pools to store fertilizer N; (2) Inhibitor application improves retention of urea-N through abiotic process in the absence of additional C; (3) Inhibitor application with straw alleviates the decrease of N uptake and crop yield caused by straw because of the persistence of NH_4_^+^ under the combined application of inhibitors.

## Results

### Total soil N and retention of urea-N

Total soil N did not show any significant differences with either application of inhibitors or straw return in the current research (*P* > 0.05). Only the combined application of inhibitors and straw (UIS) increased soil total N at the rice maturation stage compared with the other three treatments (Table 1). In contrast, different treatments and sampling times showed significant effects on recovery of urea-N (Table 1). UI decreased N recovery rate by 18.3%, 28.4%, and 39.4% for urea-N at the maturation stage compared with U, US and UIS (*P* < 0.05). Straw return promoted the retention of urea-N in soil, especially at the tillering stage, which is the rice growth period during which most N is taken up. Combined application of inhibitors and straw (UIS) alleviated the retention of urea-N in soil and decreased urea-N recovery rate by 41.29% compared with US at the tillering stage.

**Table 1:** Soil total nitrogen, soil urea-derived nitrogen and the recovery of urea-N in soils at three sampling stages.

Treatment	Seedling stage			Tillering stage			Maturation stage		
	Soil nitrogen (g kg^−1^)	Soil urea-derived nitrogen (mg kg^−1^)	Recovery rate (%)	Soil nitrogen (g kg^−1^)	Soil urea-derived nitrogen (mg kg^−1^)	Recovery rate (%)	Soil nitrogen (g kg^−1^)	Soil urea-derived nitrogen (mg kg^−1^)	Recovery rate (%)
U	0.99 ± 0.04Aa	44.9 ± 9.4ABa	42.80Aa	0.97 ± 0.01 Ab	26.3 ± 3.25Bb	18.17Bb	1.03 ± 0.05 Aa	51.9 ± 6.78Abc	27.44Bbc
US	1.03 ± 0.05Aa	35.2 ± 6.41Bab	33.58Bab	1.03 ± 0.01 Aa	66.5 ± 4.18Aa	45.53Aa	1.03 ± 0.02 Aa	56.2 ± 7.56Aab	31.31Bab
UI	1.01 ± 0.01Aa	27.8 ± 3.41Bbc	26.56Abc	1.02 ± 0.05 Aab	26.5 ± 4.03Bb	18.32Bb	1.02 ± 0.05 Aa	46.2 ± 5.83Ac	22.42ABc
UIS	1.08 ± 0.03Aa	19.4 ± 3.74Bc	18.57Bc	1.01 ± 0.03 Aab	38.9 ± 8.88Bb	26.73ABb	1.06 ± 0.02 Aa	63.8 ± 2.41Aa	37.01Ba

U: ^15^N labeled urea; US: ^15^N labeled urea + straw; UI: ^15^N labeled urea + inhibitor; UIS: ^15^N labeled urea + inhibitor + straw. The values in the table represent the mean of three replicates. Different capital letters represent significant differences of the same treatment at different sampling times; different lowercase letters represent significant differences among treatments during the same sampling period (Duncan, *P* < 0.05).

### Dynamics of soil N and fate of urea-N in NH_4_^+^-N, MBN, FA, DON pools

**Figure 1:**
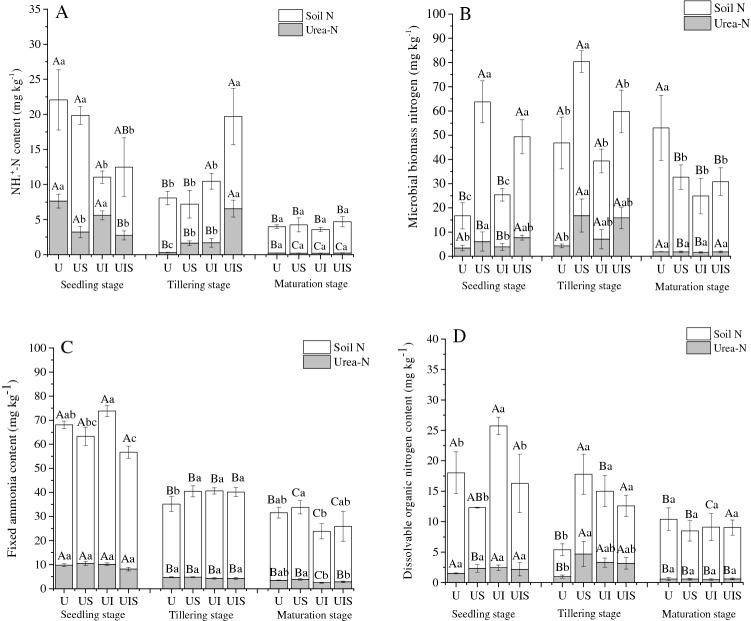
Soil and urea-derived nitrogen with straw, inhibitor and nitrogen fertilizer application at three sampling stages. U: ^15^N labeled urea; US: ^15^N labeled urea + straw; UI: ^15^N labeled urea + inhibitor; UIS: ^15^N labeled urea + inhibitor + straw. (**A**) Ammonium (NH_4_^+^-N), (**B**) Microbial biomass nitrogen (MBN), (**C**) Fixed ammonium (FA), (**D**) Dissolved organic nitrogen (DON). Different capital letters represent significant differences of the same treatment at different sampling times; different lowercase letters represent significant differences among treatments during the same sampling period (Duncan, *P* < 0.05).

**Figure 2:**
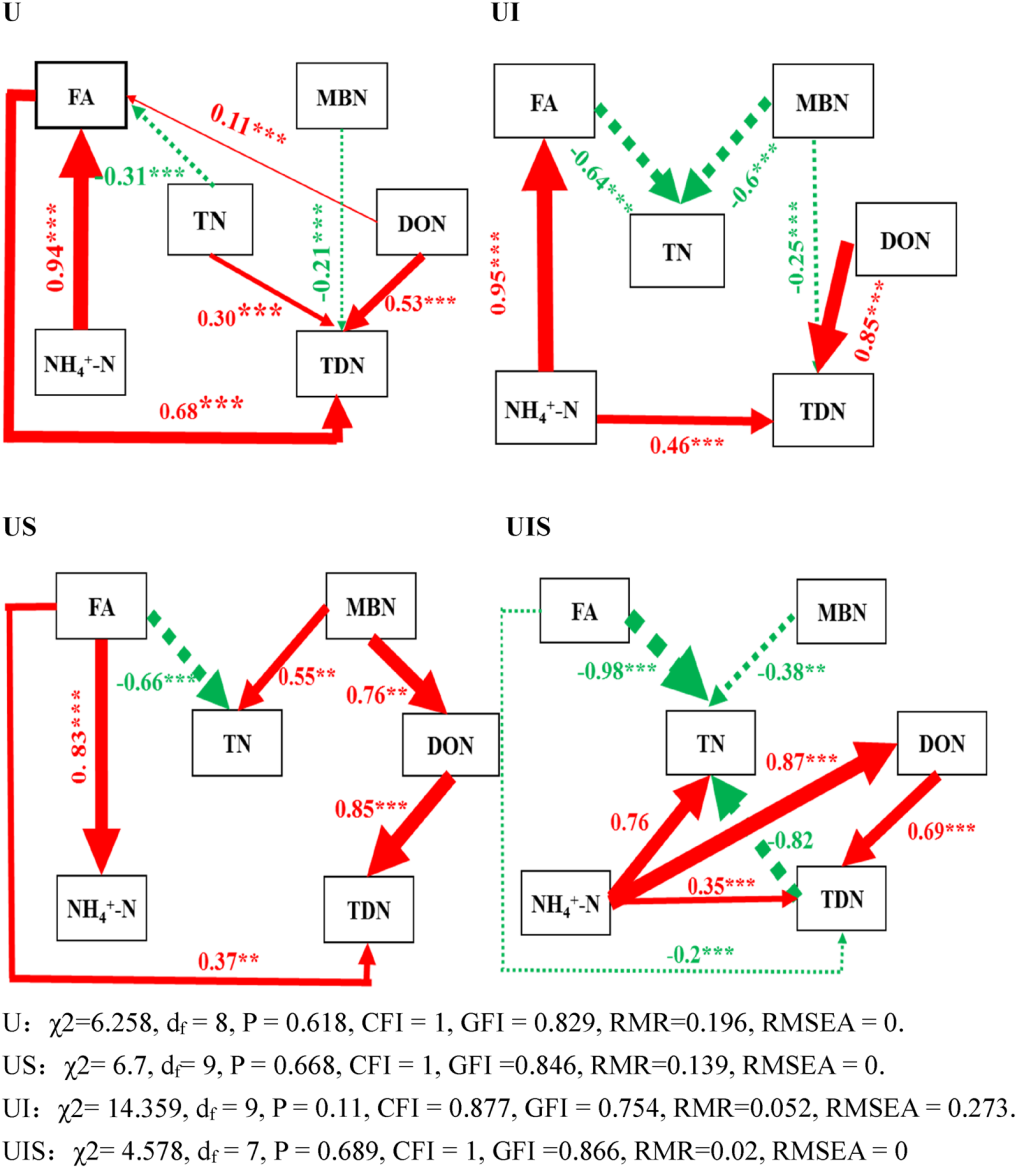
Structural equation model analysis for the contribution of urea-N to soil total nitrogen retention. The numbers on the arrows are normalized path coefficients. The thickness of the arrow is proportional to the size of the path coefficient. Continuous arrows and dotted arrows indicate positive and negative correlations, respectively. Asterisks after data indicates a significant difference (*P < 0.05, **P < 0.01, *** *P* < 0.001). U: ^15^N labeled urea; US: ^15^N labeled urea + straw; UI: ^15^N labeled urea + inhibitor; UIS: ^15^N labeled urea + inhibitor + straw. FA stands for fixed ammonium derived from urea; MBN stands for microbial biomass nitrogen derived from urea. DON represents soluble organic nitrogen derived from urea; TN represents total nitrogen derived from urea sources; NH_4_^+^-N denotes ammonium nitrogen derived from urea. TDN represent total dissolvable nitrogen derived from urea. Some variables have no arrows because in the operation of maximum likelihood number method, the route with relatively small path coefficient is removed in order to make the model more fit and more in line with the fitting index described below in the figure.

In general, urea-derived NH_4_^+^-N decreased with rice growth for all treatments except UIS, which increased initially and then decreased. At the seedling stage, after 7 days of fertilization, U and UI treatments showed a significant higher ^15^NH_4_^+^ content than US and UIS treatments (*P* < 0.05). Compared with urea only (U), urea-derived NH_4_^+^ in US, UI and UIS significantly decreased by 57.8%, 26.5% and 64.5%, respectively, at the seedling stage (*P* < 0.05) (Fig. 1A). At tillering, just after the top dressing fertilization, the lowest amount of fertilizer-derived NH_4_^+^ was found in the U treatment while the highest recovery rate was found in the UIS treatment, which was 20 times higher than the U treatment and 23.5% higher than the US treatment (Fig. 1A, Table 2). There were no significant differences in NH_4_^+^ and ^15^NH_4_^+^ content at the end of plantation among treatments (Fig. 2, Table 2, *P* > 0.05).

**Table 2:** Recovery of fertilizer nitrogen in each soil nitrogen pool (%).

	NH_4_^+^-N	FA	MBN	DON	TDN
**Seedling**					
U	7.39Aa	9.34Aa	1.77Ab	1.42Aa	3.79Aa
US	3.12Ac	9.98Aa	4.49Ba	2.21Aa	5.06Aa
UI	5.43Ab	9.72Aa	2.42Bb	2.32Aa	3.96Aa
UIS	2.66Bc	7.80Aa	5.45Ba	2.06Aa	3.95Aa
**Tillering**					
U	0.21Bb	3.17Ba	1.49Ab	0.65Bb	1.63Bc
US	1.11Bb	3.20Ba	11.59Aab	3.11Aa	5.08Ab
UI	1.14Bb	2.87Ba	12.4Aa	2.19Aab	12.41Aa
UIS	4.45Aa	2.83Ba	12.9Aa	2.11Aab	3.85Ab
**Maturation**					
U	0.15Ba	2.31Bab	1.91Aa	0.38Ba	0.80Ba
US	0.14Ca	2.60Ba	4.61Aa	0.38Ba	1.16Ba
UI	0.13Ca	1.66Bb	1.79Ba	0.32Ba	0.66Ba
UIS	0.16Ca	1.91Bb	4.49Ba	0.40Ba	0.98Aa

U: ^15^N labeled urea; US: ^15^N labeled urea + straw; UI: ^15^N labeled urea + inhibitor; UIS: ^15^N labeled urea + inhibitor + straw. The values in the table represent the mean of three replicates. NH_4_^+^-N: ammonia nitrogen derived from urea; FA: fixed ammonia derived from urea; MBN: microbial biomass nitrogen derived from urea; DON: dissolved organic nitrogen derived from urea; TDN: total dissolved nitrogen derived from urea. Different capital letters represent significant differences of the same treatment at different sampling times; different lowercase letters represent significant differences among treatments during the same sampling period (Duncan, *P* < 0.05).

Soil microbial biomass N (SMBN) and fertilizer-derived N in microbial biomass are shown in Fig. 1B; uptake by microbial biomass is a biotic process by which soil can retain the added N. The treatment effect was stronger than the effect of sampling stage for this parameter (Fig. 1B). It is apparent that, compared with U, straw addition (US, UIS) significantly increased the recovery of urea-N in the microbial biomass N pool, as the urea-derived MBN increased 77.9% and 122.7% in the seedling stage and 191.9% and 269.6% at tillering (Fig. 1B, Table 2). At maturation, however, there was no significant difference among treatments for MBN and MB^15^N, except that the U treatment exhibited 20.35–27.92 mg N kg^−1^ higher MBN than other three treatments (Fig. 1B). It is emphasized that MB^15^N was highest with the application of rice straw (US, UIS), which constrained the supply of fertilizer N to rice growth. Compared with U, inhibitor application did not show a significant influence on MBN and MB^15^N except that U exhibited higher MBN at maturation compared with the other three treatments (Fig. 1B, Table 2).

The fixed ammonium (FA) pool is correlated with abiotic process. Generally, both total soil FA and urea-derived FA exhibited the pattern of seedling > tillering > maturation (*P* < 0.01, Fig. 1C), with the highest FA recovery of 10% at the seedling stage (Table 2), indicating that the soil had a strong ability to fix N at first and this pool was released thereafter. At seedling, UI showed the highest FA and the treatments without straw (U, UI) had higher FA contents 9.8% and 31.1% than that in the treatments receiving straw (US, UIS). Soil FA decreased sharply to 12.61–27.87 mg N kg^−1^ at tillering for all treatments and U exhibited the lowest value with 30.42 mg N kg^−1^. FA decreased further at maturation stage except in the U treatment. The amount of urea-derived N conserved in FA did not show any significant difference at seedling and tillering in different treatments (*P* > 0.05), and at maturation, the addition of inhibitors (UI, UIS) led to greater release of urea-derived FA and showed lower values compared with U and US (Fig. 1C).

Another N pool which is correlated with both abiotic and biotic process is dissolved organic N (DON). The recovery of urea-derived DON was not significantly different among treatments at all three sampling periods, except the US treatment at tillering, which increased 3.82 times relative to U, reaching 4.67 mg N kg^−1^ (*P* > 0.05, Fig. 1D, Table 2). For total DON, different treatments showed contrasting changes. US decreased at the seedling stage and increased at tillering compared with U, and UI increased significantly at both seedling and tillering. UIS changed only at tillering stage, which increased 2.38 times compared with U (Fig. 1D).

### Rice yield and N uptake under the application of inhibitors and straw

According to Table 3, rice yield and biomass in the US treatment was significantly reduced compared with the other treatments, and the loss of urea-N was significantly higher than the other treatments. Inhibitor addition together with straw (UIS) alleviated the reductions in yield and rice biomass caused by straw only (US). These two treatments with straw application permitted greater retention of urea N in the MBN pool. Compared with C, the U, UI and UIS treatments significantly increased rice biomass by 20.0%, 24.7% and 19.8% respectively (*P* < 0.05), and rice yield increased by 8.2%, 21.9% and 16.3%, respectively. Furthermore, the UI treatment achieved the highest rice yield, biomass, panicle N content and total fertilizer N uptake with only 30% fertilizer N loss.

**Table 3:** Rice yield, biomass, nitrogen content assimilated from urea and the fate of urea-derived N at harvest (% of applied N).

Treatment	Yield (g)	Biomass (g)	Panicle N uptake (g)	Rice N uptake (g)	Rice urea N uptake (%)	Soil urea-N retention (%)	Unaccounted-for (%)
C	13.5 ± 2.47c	43.2 ± 4.57c	5.6 ± 0.82d	6.2 ± 0.87d	–	–	–
U	20.5 ± 0.43ab	67.2 ± 2.18a	147.4 ± 3.60ab	231.6 ± 13.40b	51.4 ± 0.05b	11.5 ± 0.02a	36.9 ± 0.04b
US	18.9 ± 1.45b	56.0 ± 7.75b	122.4 ± 9.54c	173.3 ± 31.97c	38.5 ± 0.11c	12.5 ± 0.02a	48.9 ± 0.11a
UI	23.1 ± 1.61a	69.9 ± 4.13a	157.6 ± 8.94a	273.1 ± 27.23a	60.7 ± 0.04a	10.2 ± 0.01a	29.0 ± 0.05c
UIS	22.0 ± 1.71ab	67.1 ± 3.25a	143.3 ± 5.10b	228.6 ± 4.99b	50.8 ± 0.06b	14.2 ± 0.01a	34.9 ± 0.05b

C: no nitrogen, inhibitor and straw; U: ^15^N labeled urea; US: ^15^N labeled urea + straw; UI: ^15^N labeled urea + inhibitor; UIS: ^15^N labeled urea + inhibitor + straw. The values in the table represent the mean of three replicates. Different lowercase letters represent significant differences among treatments during the same sampling period (Duncan, *P* < 0.05).

## Discussion

### Effect of straw return on the fate of urea-N

Hypothesis 1 was confirmed by the current research. The lowest N uptake and crop yields were found in the US treatment (Table 3), which is in accordance with previous research conducted by Pan et al.^17^ and Ma et al.^21^. In irrigated rice, grain yield is closely associated with N uptake when the availability of other nutrients is adequate and pest damage does not limit crop growth. Straw decomposition and utilization by microorganisms led to a state of N limitation in the soil due to the high C/N ratio of straw, and microorganisms competed for N with plants, especially in the critical period of crop growth^21^. The immobilization of urea-N (MBN) was enhanced by 2.53, 7.78 and 2.41 times, respectively, at all three sampling stages compared with U (Fig. 1B), and there were significant correlations among urea-derived MBN and DON (*P* < 0.01, Fig. 2), in which straw return showed the main effect on soil MBN and DON at seedling and tillering (*P* < 0.05) and sampling time had a significant influence (*P* < 0.05, Table 4). This is likely one of the reasons for the decrease in crop yield and N uptake. Devevre and Horwath^22^ indicated that in addition to microbially mediated biotic process, chemically mediated abiotic process are also responsible for the retention and supply of fertilizer N in paddy soil systems. They postulated that fertilizer-derived organic nitrogen accumulated in soil undergoes partial polymerization and enters the soil humus, resulting in the decrease in N availability. We observed that urea-derived DON was significantly higher at the earlier stages of sampling in the current study. Although we did not measure SOM fractions, we assumed the increased DON would help form stabilized humic substances with the help of C existing in straw. Olk et al.^23^ also suggested that the accumulation of recalcitrant rice straw products, such as lignin-derived phenols, was responsible for reducing soil N availability. They theorized that stabilization of N into humic fractions might be the dominant process affecting the availability of fertilizer N under the combined application of straw. Despite the immediate negative effect of straw application for rice growth and N uptake, the long-term effects of N retention induced by plant residue addition appear to be positive. Bird et al.^24^ examined the effects of residue management on N immobilization and uptake by rice plants. They observed that although N fertilizer efficiency fell after the return of rice straw in the short term, 4–6 years of straw addition produced a labile pool of available N, which led to a reduction in N fertilizer dependency by the rice plant in subsequent seasons.

**Table 4:** Interactive function between treatments and sampling times on nitrogen derived from soil and urea in different nitrogen pools based on the two-way ANOVA analysis.

	STN	SNH_4_^+^	SFA	SMBN	SDON	STDN	UTN	UNH_4_^+^	UFA	UMBN	UDON	UTDN
Treatment	***	***	***	***	***	***	***	***	***	***	***	***
Sampling times	N	*	N	***	**	N	***	***	N	*	***	**
Sampling time* treatment	N	***	**	***	**	***	***	***	N	N	**	**

STN: Soil total nitrogen; SNH_4_^+^: Soil ammonia nitrogen; SFA: Soil fixed ammonia; SMBN: Soil microbial biomass nitrogen; SDON: Soil dissolved organic nitrogen; STDN: Soil total dissolvable nitrogen; UTN: Urea-derived total nitrogen; UNH_4_^+^: Urea-derived ammonia nitrogen; UFA: Urea-derived fixed ammonia; UMBN: Urea-derived microbial biomass nitrogen; UDON: Urea-derived dissolved organic nitrogen; UTDN: Urea-derived total dissolvable nitrogen.

*P < 0.05; **P < 0.01;***P < 0.001; N: *P* > 0.05.

It should be noted that a large part of urea N was fixed in the form of FA, and FA decreased significantly over time, indicating that the release of FA can contribute to the uptake of N by rice (Fig. 1C, Table 2). This result was not in agreement with Gouveia and Eudoxie^25^ and Akter^26^, who indicated that under anaerobic conditions, the surface of clay minerals was covered by iron oxides, which limited the diffusion of NH_4_^+^ into the interlayer space, weakening NH_4_^+^ fixation by clay minerals. The reason for the discrepancy can be attributed to the variety of parent materials in different soil types. Alfisols originate from residual deposits, slope deposits and some loessial materials with more montmorillonite and illite, abundant 2:1 clay minerals that favor the fixation of NH_4_^+^ in soil^27^. In the present experiment, structural equation modeling also indicated that extractable NH_4_^+^ was significantly correlated with FA (the pathway coefficient was 0.83, *P* < 0.01) (Fig. 2 US). Recently, several researchers have indicated that FA is an important N pool for the retention of urea-N in soils similar to that used here^7,27^, and the newly formed FA can be later released and afford available N for plant growth^28,29^. The current study confirmed this process. Other research has indicated that with addition of glucose, competition for NH_4_^+^ between microbial immobilization and mineral fixation was intensified and a larger proportion of urea-N was found in the organic N pool, reducing the effect of FA on urea-N conservation^21,30^. In our experiment, however, the decrease in FA did not necessarily exhibit any relationship with MBN (Fig. 2); other research has also shown that the addition of wide C/N ratio organic material does not necessarily decrease FA^31^. In addition to the various types of soils tested and their mineral composition^32^, the discrepancies among the studies were induced by differences in rate and availability of the C addition^33^. The current research emphasized the importance of NH_4_^+^ fixation in the early season and N release later in the season for the growth of rice. Reserves of N in FA can be a substantial source of N for plant uptake and other soil processes, and should be given greater attention in future work.

The N unaccounted for in the US treatment was significantly higher than in other treatments (Table 3), which is in disagreement with previous research^3,22^, but in accordance with the research conducted by Phongpan and Mosier^34^ and Wang^35^. The contrasting results from different trials are probably attributable to differences in soils and different C/N of straw. Loss of N through surface runoff did not occur in this study, and the pot experiment also avoided losses through percolation^3^. Therefore, ammonia volatilization and gases produced during nitrification–denitrification are responsible for the N loss^36^. Zaman and Blennerhassett^37^ indicated that decomposition of the incorporated straw triggered the release of base cations as well as increased floodwater pH, which promoted NH_3_ volatilization. Moreover, total dissolved nitrogen (TDN) and urea-derived TDN was higher in the US treatment (data not shown), which increased the risk of N volatilization loss through the water, and is probably the reason why unaccounted-for N is higher in the US treatment. Zhong (2017)^38^ indicated that the percentage of N loss by ammonia volatilization increased with the nitrogen application rate. 48.77% loss as ammonia volatilization was reported when 225 kg N ha^−1^ was applied while 58% loss was found when 375 kg N ha^−1^ was applied. We did not measure ammonia volatilization in the current research, but still can speculate that the volatilization loss of N was lower than the experiment which applied more N to soil.

### Fate of urea-N under the application of inhibitors

Application of inhibitor with urea (UI) showed the highest rice yield and N uptake as well as lowest unaccounted-for N among all the treatments (Table 3). Nitrification and urease inhibitors have been proposed as a method to improve N use efficiency and reduce N losses^39,40^. Although the response of crop yield to inhibitors is variable, a meta-analysis concluded that the addition of inhibitors caused yield and N use efficiency to increase by 7.5% and 12.9%, respectively^19,29,41^, which is in accordance with our research. Zhang et al.^42^ and Tobias et al.^43^ indicated that urea hydrolysis can be delayed 3–7 days with the application of urease inhibitor. At the same time, nitrification inhibitors regulate the existing forms of extractable N, allowing a better synchrony between N supply and rice demand^44^. In our experiment, we observed relatively high urea-derived NH_4_^+^ at the seedling stage in the presence of inhibitor, and a decrease at tillering, but the decrease was not as strong as in the U treatment (Fig. 1A), which confirmed the regulatory function of inhibitors as well. This is beneficial for rice uptake of N to promote higher yield. MBN in UI was not as strong as that in the treatments with application of straw (US and UIS) at seedling and maturation (Fig. 1B). This phenomenon can be attributed to the widespread view that there is less available C supply with the application of straw^7^. FA showed the same pattern of change as the other treatments, showing the highest value at seedling which decreased sharply over time (Fig. 1C). Compared with other treatments, the proportion that decreased is higher, indicating that newly fixed ammonium was more easily released in this treatment. According to the SEM analysis shown in Fig. 2, the urea-derived NH_4_^+^ had a significant direct pathway to FA (the pathway coefficient was 0.95, *P* < 0.01). This phenomenon verified hypothesis 2, but only at the seedling stage of rice growth.

The recovery rate of urea-N in soil was significantly lower in UI than in other treatments, and loss (unaccounted-for N) was lower as well, but uptake of urea N by plants was significantly higher (Tables 1, 3). Basically, urea-N can be partitioned into three fates after it is applied to soil: retained in soil, taken up by plants and lost from the soil–plant system. As mentioned earlier, the possible loss pathways in this study research are ammonia volatilization and nitrification–denitrification. Although a lot of work has indicated increased ammonia volatilization under waterlogged conditions of paddy soil with the application of NI^45,46^; our experiment still showed a decreased N loss. This can likely be attributed to decreased loss of gases during nitrification and denitrification. More importantly, application of inhibitor made urea-N transformation characteristics better match the demand of rice growth. The advantages of inhibitor amendment were demonstrated in the current research, i.e. increased rice yield and decreased N loss, which implies better protection of the environment. But the disadvantage of inhibitor amendments was also evident: the lower retention of fertilizer N in soil may cause a stronger dependence on fertilizer N in rice-soil systems for ensuring favorable long-term yields.

### Fate of urea-N under application of inhibitors and straw

The UIS treatment exhibited significantly higher urea-N recovery compared with UI or any other treatment at maturation, and was also higher than UI at tillering (Table 1), which indicated the good effect of combined application of straw on N retention and soil fertility improvement, where a positive interaction was evident between them on soil N retention at tillering (*P* < 0.05). Yu et al.^27^ also indicated that in the presence of glucose, microbial immobilization was the principal mechanism of fertilizer N retention, and this phenomenon was mitigated by urease inhibitor. Our results were also consistent with Ma et al.^7^, who inferred that the application of straw together with inhibitor promotes fertilizer N retention in soil compared with inhibitor application alone. This is in contrast to the results of Wang et al.^47^, who observed that inhibitors decreased N immobilization when soil was amended with barley straw. The difference may be attributed to the contrasting C/N ratio of straw, different soil type and planting system, and the fertilizer regimes; according to Table 4, sampling times had a significant influence on recovery of urea-N in soil pools except for FA. When compared with US, UIS alleviated the decrease in crop yield and N uptake of urea-N (Table 3), which verifies the proposed hypothesis 3, and is in accordance with the results published by Ma et al.^21^. He proved that it is urease inhibitor, not nitrification inhibitor, which alleviates the decrease in crop yield induced by straw application. The delayed effect of urease inhibitor for appearance of NH_4_^+^ (and presumably reduced NH_3_ emission) in UIS gave rice roots more opportunity to catch and absorb N, which led to higher uptake of N and higher crop yield^21,48^. FA showed the same pattern as the other treatments (Fig. 1, Table 2), which also emphasizes the importance of FA in the earlier growth stage and release of FA in the later growth stage for the retention and supply of N in soil^30,49^. Future experiments, particularly field experiments, will further test this effect.

## Materials and methods

### Soil collection

Soil samples were obtained from a paddy field to a depth of 20 cm at the University of Shenyang Agriculture Rice Institute Experiment Site, Northeast China (41°31′ N, 123°24′ E), which had received no fertilizer in the past 20 years. The soil type was an Alfisol before it was changed to rice cultivation and represents the main soil type for agricultural production in the region. The field-moist soil was passed through a 5 mm sieve to remove stones, crop residues and roots. The basic soil physical and chemical properties were as follows: the pH (H_2_O) was 6.18, soil organic matter content was 26.1 g kg^−1^, total N content was 1.95 g N kg^−1^, total P and K were 2.18 g kg^−1^ and 22.4 g kg^−1^ respectively. Soil CEC was 22.3 cmol kg^−1^, and clay, silt and sand were 16.5%, 55.3% and 28.2% respectively.

### Set-up

An outdoor pot experiment was conducted with rice in a net house from May to October, 2018. Five treatments were set up in a completely randomized design: (1) C (no nitrogen); (2) U (^15^ N-labeled urea); (3) US (^15^urea + rice straw); (4) UI (^15^urea + urease inhibitors + nitrification inhibitor); (5) UIS (^15^urea + urease inhibitors + nitrification inhibitor + rice straw). A total of 45 pots (5 treatments × 3 sampling times × 3 replicates) were used, with 3 kg air dried soil per pot. The ^15^N-labelled urea (10.02% ^15^N at%) was applied at a rate equivalent to 318 kg N ha^−1^ in three applications: 50% as a basal application, and 25% each as the first and second top dressing. Super-phosphate calcium and potassium chloride were applied at a rate of 212 kg P ha^−1^ and 318 kg K ha^−1^ respectively. Urease inhibitors (PPD- phenylphosphodiamine and NBPT) and nitrification inhibitor (DMPP) were applied at rates of 1%, 1% and 2% of applied urea-N (w/w). Rice straw with a C/N ratio of 63 (C and N contents were 37.8% and 0.59%, respectively) was ground into powder with a pulverizer. The application amount of straw was 10.6 t ha^−1^ (5 g kg^−1^). The basal fertilizer (with or without inhibitor), straw and soil were mixed thoroughly into the pots on May 25 and left overnight under flooded conditions. Rice was transplanted into pots the next day. Fertilizer (with or without inhibitor) was dissolved in water and injected into the soil with a syringe when topdressing on June 18 and August 20.

Rice (Meifeng 9) with five or six fully expanded leaves was grown in plastic pots (d = 18 cm, h = 25 cm) under no-leached conditions. Crop management was consistent with local management practices for conventional cultivation, in particular maintaining 3–5 cm floodwater until 1 week before harvest. Soil and rice plants were sampled with a five-point method after 7 days fertilization at the seedling stage (June 4), and then at the tillering (June 25) and maturation stage (September 21).

### Soil analysis

Soil pH was determined by a glass electrode pH meter using a soil:water ratio of 1:2.5^50^, Total organic C content of soils was determined using a C and N analyzer (Vario TOC Analyzer, Elementar, Germany). Soil total P and K was determined by sodium carbonate fusion and the molybdenum antimony–ascorbic acid colorimetric method^51^. The CEC was determined using a modified NH_4_-acetate compulsory displacement method^52^. Soil particle size distribution was determined by pipette method^41^. Ammonium was determined by extracting a 10 g soil subsample with 100 mL of 2 M potassium chloride (KCl). Samples were shaken for 1 h on a reciprocal shaker, filtered and analyzed on a continuous flow analyzer (AA3, Bran + Luebbe, Germany)^53^. Microbial biomass N was determined by the chloroform fumigation extraction method^54^. Briefly, 20 g of moist soil were fumigated with ethanol-free chloroform for 24 h. The fumigated and a non-fumigated control sample were both extracted with 80 mL of 0.5 M potassium sulfate (K_2_SO_4_) on a reciprocal shaker for 30 min before filtering. The concentration of extractable N in both samples was determined on a C and N analyzer (Vario TOC Analyzer, Elementar, Germany). To calculate MBN, the difference in extractable N content between the fumigated and non-fumigated samples was divided by 0.54 to account for incomplete extraction^54^. Fixed ammonium (FA) was determined by the KOBr-KOH method^55^ and soil total FA was titrated with standard acid after distillation by Kjeldahl method^56^. Total dissolved nitrogen (TDN) was analyzed with alkaline persulfate oxidation method of Cabrera and Beare^57^. Dissolved organic N (DON) was calculated by subtracting inorganic N from TDN^14^. Total soil N was analyzed with an elemental analyzer after the soil was air-dried and ball-milled to a fine powder. Three pots of crop were taken at maturation stage to calculate biomass and then separated to stem and panicle to get yield and panicle N uptake. Cleaned rice plant samples were oven-dried at 65 °C to constant weight and ground for analysis of total N analysis and ^15^N content.

The atom% ^15^N of the NH_4_^+^ pool was determined as described by Sebilo et al.^58^. Briefly, the extracted solutions were first transferred to glass vials with caps. Filter packages for the NH_4_^+^ diffusion technique were prepared as follows: KHSO_4_ (10 μL, 2.5 M) was pipetted onto a strip of glass fibre filter (APFE, 25 mm, Millipore), and the filter was then wrapped and enclosed with a hydrophobic filter (“Mitex”, PTFE, 47 mm diameter, Millipore) to form the filter package. After the addition of MgO (0.25 g) and the filter package to the samples, the vials were closed immediately and the solution was stirred slowly for one week at room temperature. After one week, the filter package was removed from the vial, dried in a freeze dryer for 24 h and the glass fibre strip recovered for atom% ^15^N analysis. For measurement of ^15^MBN, 4 mL fumigated and unfumigated subsamples were digested to transform N to NO_3_^−^^53^, then ^15^NO_3_^−^ was measured with 0.25 g devarda alloy reagent (Merck, German) and a diffusion package method as described in Sebilo et al.^58^. The digested unfumigated glass fiber samples were considered as ^15^TDN, and ^15^DON was calculated by subtracting inorganic N from ^15^TDN. In order to measure ^15^FA, the Kjeldahl sample was oven-dried to crystal at 70 °C and prepared for further determination. For all samples (including plant, soil and fiberglass), ^15^N content was determined by a Stable Isotope Ratio Mass Spectrometer (253 MAT, Thermo Finnigan, Germany).

### Statistical analysis

All statistical analyses were performed with Excel and SPSS 16.0, using the Duncan method at a significance level of 0.05 with One-way ANOVA and Two-way ANOVA. Data are presented as means of three replicates. Structural Equation Modeling (SEM) Amos 7 was used to evaluate the contribution of urea-derived N to NH_4_^+^-N, FA, MBN, DON and TDN regarding soil retention and supply with straw or inhibitor inputs. Figures were generated using the Origin 8.0 program.

The amount of urea-derived N in a certain N pool was calculated as follows^27^:

$${\text{Ndfu}}\,\left( {{\text{mg}}\,{\text{kg}}^{{ - 1}} } \right)\, = \,\frac{{{\text{N}}~_{{soil\,N\,pool}} \, \times \,^{{15}} {\text{N}}~{\text{AT}}\% ~excess_{{soil\,N\,pool}} }}{{^{{15}} {\text{NAT}}\% \,excess_{{urea}} }}$$

where Ndfu is the amount N derived from urea; N_soil N pool_ refers to the N content from different soil N pools; ^15^N AT% excess_soil N pool_ is the ^15^N enrichment of different soil N pools; ^15^N AT% excess_urea_ refers to the enrichment of urea added.

Recovery of urea-N in a specific N pool was calculated as follows:

$$\mathrm{Recovery}({\%})=\frac{{\mathrm{N}}_{dfu}}{\mathrm{ N \,urea\, applied}}\times 100$$

where Ndfu is the amount N derived from urea; N urea applied is the content of urea applied.

Uptake of urea-N by rice (N _r_) was calculated as follow:

$${\mathrm{N}}_{{\mathrm{r}}} \, = \,{\mathrm{N}}_{{{\mathrm{total}}\,{\mathrm{in}}\,{\mathrm{rice}}}} \, \times \,\frac{{\% \,atom\, excess\,15N\, in\, rice}}{{\% atom\, excess\,15N\,in\,urea}}$$

where N_total in rice_ is rice total N content; % atom excess ^15^N in rice is the enrichment of rice; % atom excess ^15^N in urea is the ^15^N enrichment of urea.
